# Overexpression of the transcription factors OCT4 and KLF4 improves motor function after spinal cord injury

**DOI:** 10.1111/cns.13390

**Published:** 2020-05-25

**Authors:** Xianpeng Huang, Chenggui Wang, Xiaopeng Zhou, Jingkai Wang, Kaishun Xia, Biao Yang, Zhe Gong, Liwei Ying, Chao Yu, Kesi Shi, Jiawei Shu, Feng Cheng, Bin Han, Chengzhen Liang, Fangcai Li, Qixin Chen

**Affiliations:** ^1^ Department of Orthopedics Surgery School of Medicine 2nd Affiliated Hospital Zhejiang University Hangzhou People's Republic of China; ^2^ Orthopedics Research Institute of Zhejiang University Hangzhou China

**Keywords:** glial scar, reprogramming, spinal cord injury, transcription factor

## Abstract

**Introduction:**

Astrogliosis and glial scar formation following spinal cord injury (SCI) are viewed as major obstacles that hinder axonal regeneration and functional recovery. Regulating the glial scar and axonal regeneration in the lesion site is important for treating SCI.

**Aims:**

Considering the important role of astrocyte in glial scar formation and subsequent axonal regeneration, we intended to investigate the effect of the transcription factors OCT4 and KLF4 on astrocyte and the underlying mechanism after spinal cord contusion injury in transgenic mice.

**Results:**

Western blotting, q‐PCR, immunofluorescence, and functional evaluation suggested that glial fibrillary acidic protein (GFAP) expression decreased in the lesion area, the porosity of the scar increased, and remyelination enhanced. Mice overexpressing the transcription factors OCT4 and KLF4 had higher Basso Mouse Scale scores than did the control mice. Moreover, using immunofluorescence and Western blotting, we discovered that some astrocytes expressed nestin and sox2 protein, suggesting that these astrocytes were reprogrammed into neural stem cell‐like cells. Furthermore, a cell scratch assay showed that the migration ability of the astrocytes was significantly inhibited in the presence of the transcription factors OCT4 and KLF4. In addition, we demonstrated that the Hippo/Yap pathway was activated after these two transcription factors overexpressed in astrocytes.

**Conclusions:**

In summary, these results suggest that overexpression of the transcription factors OCT4 and KLF4 could induce astrocyte reprogramming, which subsequently improves remyelination and functional recovery after SCI.

## 1 INTRODUCTION

Spinal cord injury (SCI) is a devastating condition that causes irreversible neuronal damage, a subsequent inflammatory response, astrogliosis, and scar formation.^1^ Glial scar formation is a complicated and dynamic process in which activated astrocytes play a significant role.^2^ Although astrocytes, microglia, pericytes, and some NG2‐positive cells all participate in the process of scar formation, hypertrophic astrocytes or activated astrocytes are indispensable and secrete extracellular matrix (ECM), mainly chondroitin sulfate proteoglycans (CSPGs).^3^, ^4^, ^5^, ^6^ Numerous studies have suggested that the glial scar not only hinders axonal regeneration but also prevents positive factors migrating into the lesion center.^7^, ^8^ Therefore, targeting the astrocyte of scar tissues could be a promising therapeutic strategy for improving functional recovery after SCI by promoting axon extension.

Various methods have been used to regulate glial scarring and axonal regeneration in recent decades, such as reactive astrocyte‐associated gene regulation, chondroitin sulfate glycosaminoglycan degradation, proteoglycan receptor inhibition, and biomaterial implantation.^9^, ^10^, ^11^, ^12^ However, these methods have limits, and the exquisite regulatory mechanism, signaling pathway network, and molecular biology behind the process of scar tissue formation and axonal regeneration are not fully understood.

Notably, since the creation of induced pluripotent stem cells (iPSCs), the cellular reprogramming associated with defined transcription factors has been used in central nervous system research.^13^, ^14^ The first direct reprogramming of fibroblasts into functional neural progenitors was performed with four reprogramming factors (OCT4, SOX2, KLF4, and c‐MYC) and opened a new door to regeneration medicine.^15^ Since this effort was initiated, transdifferentiation of cells into neurons using single transcription factors (TFs) or different combinations of TFs has also been investigated in vitro.^16^, ^17^, ^18^ Furthermore, in vivo reprogramming of astrocytes into neurons was successfully performed in central nervous system (CNS) injury using conventional reprogramming factors.^19^, ^20^, ^21^ Recently, Doeser et al reported that reprogramming mediated by the conventional reprogramming factors is capable of reducing fibrosis‐derived scars in the process of wound healing in i4F mice.^22^ These studies showed the powerful function of the OSKM (OCT4, SOX2, KLF4, and c‐MYC) TFs, which may be related to astrocyte reprogramming and indicate possibilities for astrocyte targeting after SCI. Moreover, previous studies have shown that KLF family members may regulate axonal sprouting and that OCT4 alone can induce fibroblasts into oligodendrocyte progenitor cells with substantial function.^23^, ^24^ However, the underlying mechanism of astrocyte‐associated reprogramming remains unknown, and effect of the OCT4 and KLF4 TFs on injured spinal cord has not been investigated.

Accordingly, we hypothesize that the simultaneous overexpression of OCT4 and KLF4 may induce astrocyte reprogramming and subsequently regulates axonal regeneration after spinal cord contusion injury. In this study, we used i4F mice, a mouse line that expresses OCT4 and KLF4 TFs in the spinal cord after doxycycline administration. This mouse line demonstrates more direct evidence of the effect of the “O‐K” TFs on the pathological and functional reconstitution of injured spinal cord. Our research was designed to identify a way to promote functional recovery after SCI.

## 2 EXPERIMENTAL PROCEDURES

### 2.1 Animal and surgical procedure

C57 and transgenic mice (rtTA‐OSKM mice) were obtained from the Animal Center of the Academy of Medical Science of Zhejiang Province.^25^ The Institutional Animal Care and Use Committee of Zhejiang University approved all procedures in this study. All animals were randomized to the experimental groups throughout the whole investigation. To ensure the well‐being of the animals, they had free access to food and water throughout the study, and each cage contained 3 mice. For pain management, all surgeries were carried out under deep anesthesia using pentobarbital (50 mg/kg, intraperitoneal). After anesthesia, the mice underwent T9‐T10 vertebral laminectomy. The spine was stabilized by two spinal clamps for spinal cord contusion injury. A weight drop injury of the cord was produced using a 5‐g rod (1.2 mm in diameter) dropped onto the exposed dorsal surface from a height of 20 mm (NYU impactor Model Ⅱ). After these procedures, the animals were placed in a humidity‐ and temperature‐controlled chamber. The mice were divided into 3 groups: (a) c57 mice administered doxycycline (1 mg/mL, Sigma, D9891) in drinking water (WT); (b) transgenic mice not administered doxycycline in drinking water (DOX−); and (c) transgenic mice administered doxycycline (1 mg/mL, Sigma, D9891) in drinking water (DOX+). Water‐containing doxycycline was removed after 2.5 days of administration, and fresh drinking water without doxycycline was administered for the following 4.5 days. This cycle was continued until the end of the investigation. Bladder emptying was manually performed two times daily until the end of the experiment. All animals survived the experiment, and the following inclusion criterion was enforced: mice with hindlimbs that received 0 points on the Basso Mouse Scale (BMS) locomotor scale were randomized into one of the experimental groups. A researcher blinded to the treatment group conducted this assessment. No evidence of tumor or postoperative wound infection was observed in any of the animals throughout the whole experiment.

### 2.2 Cell isolation and culture

The brains of neonatal c57 (WT) and transgenic mouse (TRANS) pups (3 days old) were removed, and cells were isolated from the cortices and cultured using a previously described procedure.^26^ When the cells reached confluence (7‐9 days), they were subjected to shaking at 200 RPM for 8 hours at 37°C to detach microglia and oligodendrocytes. Cultures obtained using this procedure were >98% astrocytes with positive GFAP staining (data not shown). After incubation for an additional 36 hours, the cells were trypsinized and reseeded in 6‐, 12‐, or 24‐well plates and maintained in 5% CO_2_ at 37°C in DMEM/F12 medium for the experimental procedures. After 3 days of culture in DMEM/F12 medium with doxycycline (2 µg/mL), one group was cultured continuously in DMEM/F12 medium with doxycycline, while the other group was reseeded in neurobasal medium (Gibco, A3582901) containing doxycycline (2 µg/mL), supplemented with 2% B27 (Gibco, A1050801), 20 ng/mL fibroblast growth factor 2 (Peprotech, 450‐33), 20 ng/mL epidermal growth factor (Peprotech, 315‐09), 10% FBS (Gibco), and 1% penicillin‐streptomycin until the end of the experiment.

The cell scratch assay was performed on confluent astrocyte monolayers cultured in DMEM/F12 medium with doxycycline. Cells were cultured in doxycycline‐containing medium (2 µg/mL), and scratches of equal sizes were created with a P200 pipette.^27^ After scratching, the cells were cultured in FBS‐free medium. The cells were photographed at day 0, 1, 2, 3, 4, and 5. Then, wound closure was measured with ImageJ software.

### 2.3 Gene expression analysis

Total RNA was extracted with RNAiso reagent (Takara). A Prime ScriptRT Reagent Kit (Takara) was applied to perform reverse transcription. All gene transcripts were intensively quantified by q‐PCR with SYBR Green (Takara) on the StepOnePlus Real‐Time PCR System (Thermo Fisher Scientific). The thermocycling conditions were as follows: 95°C (30 seconds) for initial denaturation and 40 cycles at 95°C (15 seconds) and 60°C (30 seconds).^28^ The expression of the target gene was normalized to the expression level of GAPDH rRNA and to the control group. Primers were synthesized by Sangon Biotech, and their sequences are shown below. The expression levels of the relative target genes were calculated using the 2^−ΔΔ^ *^t^* method. The following primers were used in our study:

GAPDH‐F: 5′‐TTCACCACCATGGAGAAGGC‐3′;
GAPDH‐R: 5′‐CCCTTTTGGCTCCACCCT‐3′;
Oct3/4‐F: 5′‐CTGAGGGCCAGGCAGGAGCACGAG‐3′;
Oct3/4‐R: 5′‐CTGTAGGGAGGGCTTCGGGCACTT‐3′;
Sox2‐F: 5′‐GGTTACCTCTTCCTCCCACTCCAG‐3′;
Sox2‐R: 5′‐TCACATGTGCGACAGGGGCAG‐3′;
Klf4‐F: 5′CACCATGGACCCGGGCGTGGCTGCCAGAAA‐3′;
Klf4‐R: 5′‐TTAGGCTGTTCTTTTCCGGGGCCACGA‐3′;
c‐Myc‐F: 5′‐CAGAGGAGGAACGAGCTGAAGCGC‐3′;
c‐Myc‐R: 5′‐TTATGCACCAGAGTTTCGAAGCTGTTCG‐3′.

### 2.4 Western blot analysis

Cells were washed 3 times with ice‐cold PBS and then lysed in RIPA buffer containing 1% PMSF. Total proteins were separated by SDS‐PAGE and transferred onto PVDF membranes (Millipore Sigma). After being blocked with 5% skim milk in Tris‐buffered saline with 0.1% Tween‐20 at room temperature for 1.5 hours, the membranes were incubated overnight at 4°C in Tris‐buffered saline‐Tween‐20 with antibodies specific for GFAP (ab7260, 1:2000; Abcam), oct4 (ab19857, 1:1000; Abcam), klf4 (ab129476, 1:1000; Abcam), Map2 (4542, 1:1000; CST), βⅢ tubulin (ab7751, 1:1000; Abcam), MBP (ab40390, 1:1000; Abcam), Yap1 (ab205270, 1:1000; Abcam), p‐Yap (ab76252, 1:1000; Abcam) and Erk (ab184699, 1:1000; Abcam), P‐erk (ab229912, 1:1000; Abcam), β‐catenin (ab16051, 1:1000; Abcam), and β‐actin (1:1000; Santa Cruz Biotechnology). Horseradish peroxidase‐labeled antirabbit IgG (1:5000; Santa Cruz Biotechnology) or antigoat IgG (1:5000; Santa Cruz Biotechnology) was applied as the secondary antibody for 2 hours at room temperature. Immunoreactivity was detected with ECL substrate (Millipore Sigma), and the relative protein levels were quantified by densitometry with Quantity One software (Bio‐Rad Laboratories Inc).

### 2.5 Immunofluorescence staining

Cells were fixed using 4% paraformaldehyde for 15 minutes and permeabilized and blocked with 0.05% Triton X‐100 and 2% bovine serum albumin for 30 minutes at room temperature. The fixed cells were washed 3 times with PBS and incubated overnight with anti‐GFAP (ab7260, 1:1000; Abcam), anti‐Yap1 (ab205270, 1:1000; Abcam), and anti‐p‐Yap (ab76252, 1:1000; Abcam) antibodies. The cells were incubated with an Alexa Fluor 555‐labeled secondary antibody (Beyotime: excitation and emission wavelengths of 555 and 565 nm, respectively) for 1 hour. Nuclei were stained with DAPI (Millipore Sigma; excitation and emission wavelengths of 340 and 488 nm, respectively) for 5 minutes. More than 5 microscopic fields from each sample were observed under a fluorescence microscope (DM5500; Leica). For the spinal cord, serial 5‐µm coronal and sagittal sections of the injury site were mounted on slides, washed three times in PBS, permeabilized with 0.3% Triton X‐100 for 5 minutes, and then incubated for 1 hour with 10% normal goat serum in PBS. The sections were incubated with rabbit anti‐GFAP polyclonal IgG (ab7260, 1:1000; Abcam), anti‐Neurocan (ab31979, 1:500; Abcam), anti‐Nestin (ab6142, 1:200; Abcam), and rabbit anti‐NF‐200 polyclonal IgG (ab8135, 1:500; Abcam) antibodies overnight in 5% normal goat serum in PBS at 4°C. The following day, the sections were incubated with secondary antibodies and DAPI. The results of immunofluorescence staining were quantified based on the mean fluorescence density with ImageJ software (National Institutes of Health).

### 2.6 H&E and fast blue staining

Histological analysis was performed on 45 mice. All mice were sacrificed 30 days postinjury. Following intracardiac perfusion with 0.1 mol/L phosphate‐buffered saline (PBS) and 4% paraformaldehyde in PBS, spinal cord tissue containing the lesion site was fixed for 2 hours, rinsed in 0.2 mol/L phosphate buffer overnight, cryoprotected in 30% sucrose, and frozen on dry ice. Serial transverse sections (5 µm) through the lesion site were cut with a cryostat. Hematoxylin and eosin (H&E) and immunofluorescence procedures were performed as previously described.^29^ To confirm demyelination and remyelination, the sections were rehydrated and stained with 0.1% luxol fast blue (Solarbio Science & Technology).

### 2.7 Behavioral testing

Hind limb motor function was assessed using the open‐field BMS locomotor scale system.^30^ Mice were placed into an open field and observed for 5 minutes by two independent examiners blinded to the conditions. Each day postoperation, the function of both hindlimbs was scored from 0 to 9.

### 2.8 Intralesion and lesion border quantification

To quantify the area of immunoreactivity for GFAP, NF, NEUROCAN, and Fast blue within the epicenter of the lesion, a template of the lesion area was generated in an adjacent section stained by IF/IH and placed over the proper image. MCID image analysis software (InterFocus Imaging) was used to determine the minimum signal threshold for each marker, and the total amount of labeling within the lesion was measured. The proportional area of positive labeling was determined by dividing the total target area by the lesion area and is expressed as a percentage. The amount of GFAP in the lesion border was assessed by thresholding similar to that used for analyses within the lesion. The GFAP‐positive area/whole border area (1 mm around the lesion center) was analyzed by ImageJ software.

### 2.9 Statistical analysis

All statistical analyses were performed using SPSS 21.0 software (IBM Corp.). Data were presented as the means ± SEMs (BMS score data were expressed as median ± interquartile range). An unpaired Student's *t* test was used for the comparison between two groups. One‐way ANOVA with Newman‐Keuls post hoc test was used to analyze differences in multiple comparisons. Differences in BMS scores for the groups were analyzed by nonparametric t tests. Images were analyzed by ImageJ software. All experimental groups consisted of, at least, 3 samples, and all experiments were repeated at least 3 times with independent samples. A probability level of *P* < .05 was considered significant.

## 3 RESULTS

### 3.1 Overexpression of the transcription factors OCT4 and KLF4 in the spinal cord increases the porosity of the scar tissues in the lesion site

**FIGURE 1:**
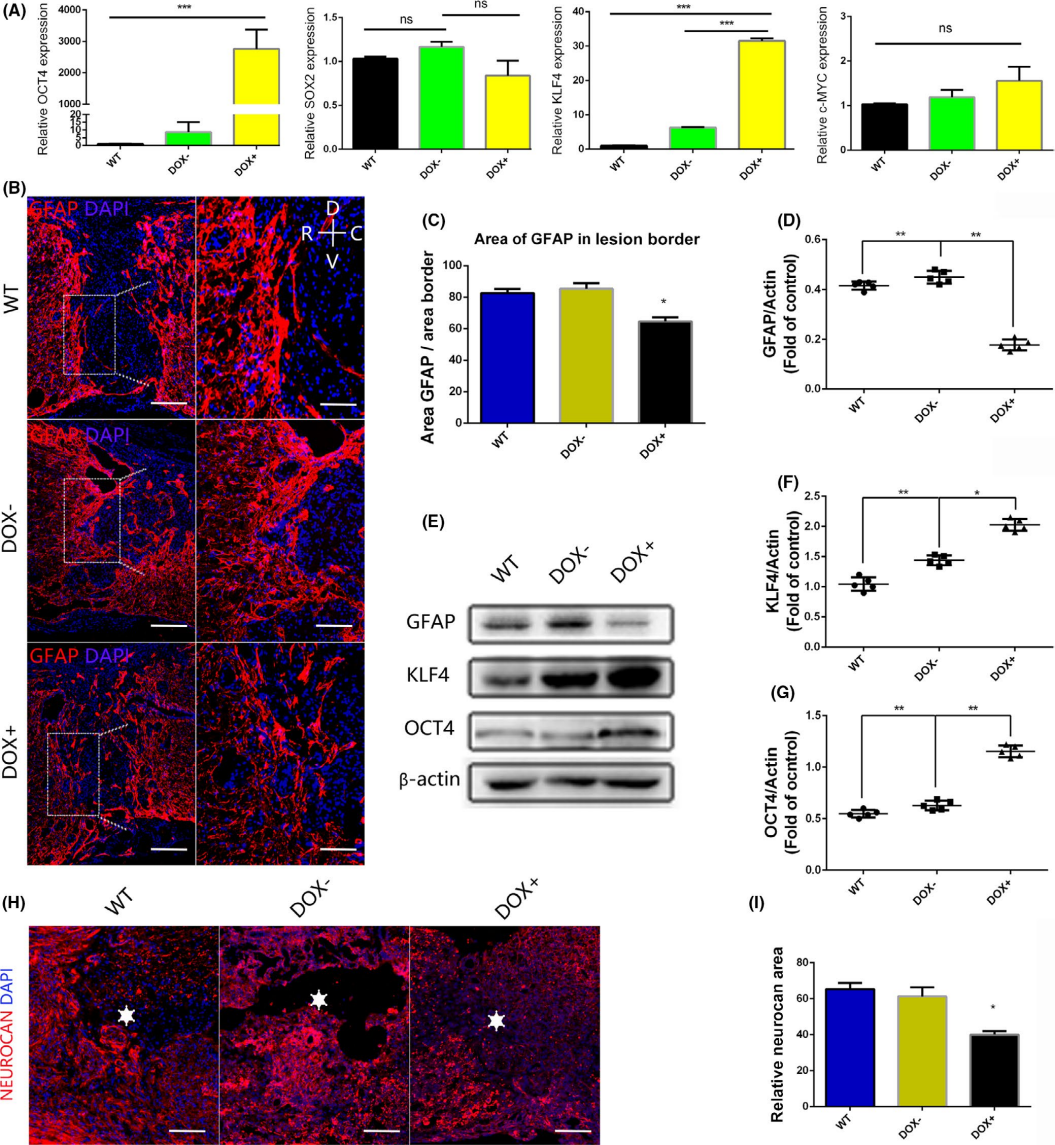
Overexpression of the transcription factors OCT4 and KLF4 increases the porosity of the scar tissues after spinal cord injury. (A) Q‐PCR results of spinal cord samples after the mice were administered water‐containing doxycycline for 3 d and normalized to GAPDH; (B) immunofluorescence staining of GFAP 4 wk after SCI, the porosity of scar was increased in DOX + group (R represents rostral, C represents caudal, V represents ventral, and D represents dorsal, scale bar, 200 or 100 μm); (C) quantification of GFAP area/border area of part (B); (D‐G) protein expression levels of OCT4, KLF4, and GFAP were measured by Western blot analysis at 4 wk after SCI; (H, I) neurocan staining of spinal cord around lesion cite at 4 wk after SCI, stars marked the lesion center, scale bar, 200 μm. Data were expressed as the mean ± SEM, one‐way ANOVA with Newman‐Keuls post hoc test, *P < .05, **P < .01, ***P < .001, n = 5 per group

In order to induce the expression of the transcription factors in vivo, we chose transgenic mouse line (rtTA‐OSKM mice), which expresses the transcription factors OCT4 and KLF4 in the spinal cord after the administration of doxycycline‐containing water for 3 days. This expression was confirmed by q‐PCR and Western blotting. Interestingly, we found that in the DOX+ group, the volume of the astrocytic scar around the lesion site was smaller than that in the other groups 4 weeks after spinal cord injury, as determined by the results of GFAP immunofluorescence, and the GFAP/border area of the DOX+ group (64.67 ± 1.45, n = 5) was lower than that of the WT group (85.33 ± 2.02, n = 5) and the DOX‐group (82.67 ± 1.45, n = 5) (Figure 1A‐C). Additionally, we analyzed the extracellular matrix, an important aspect of scar tissue secreted by reactive astrocytes. Neurocan staining demonstrated that mice administered doxycycline had fewer proteoglycans (Figure 1H,I). These data showed direct evidence of increased porosity, which promotes neuron sprouting and neurotrophic factor filtration.^31^ To confirm the GFAP protein expression level, we performed Western blotting. The GFAP protein expression level in mice overexpression OCT4 and KLF4 TFs was lower than that in WT mice, which is consistent with the immunofluorescence results (Figure 1D‐G).

### 3.2 Remyelination is improved in the presence of the transcription factors OCT4 and KLF4

**FIGURE 2:**
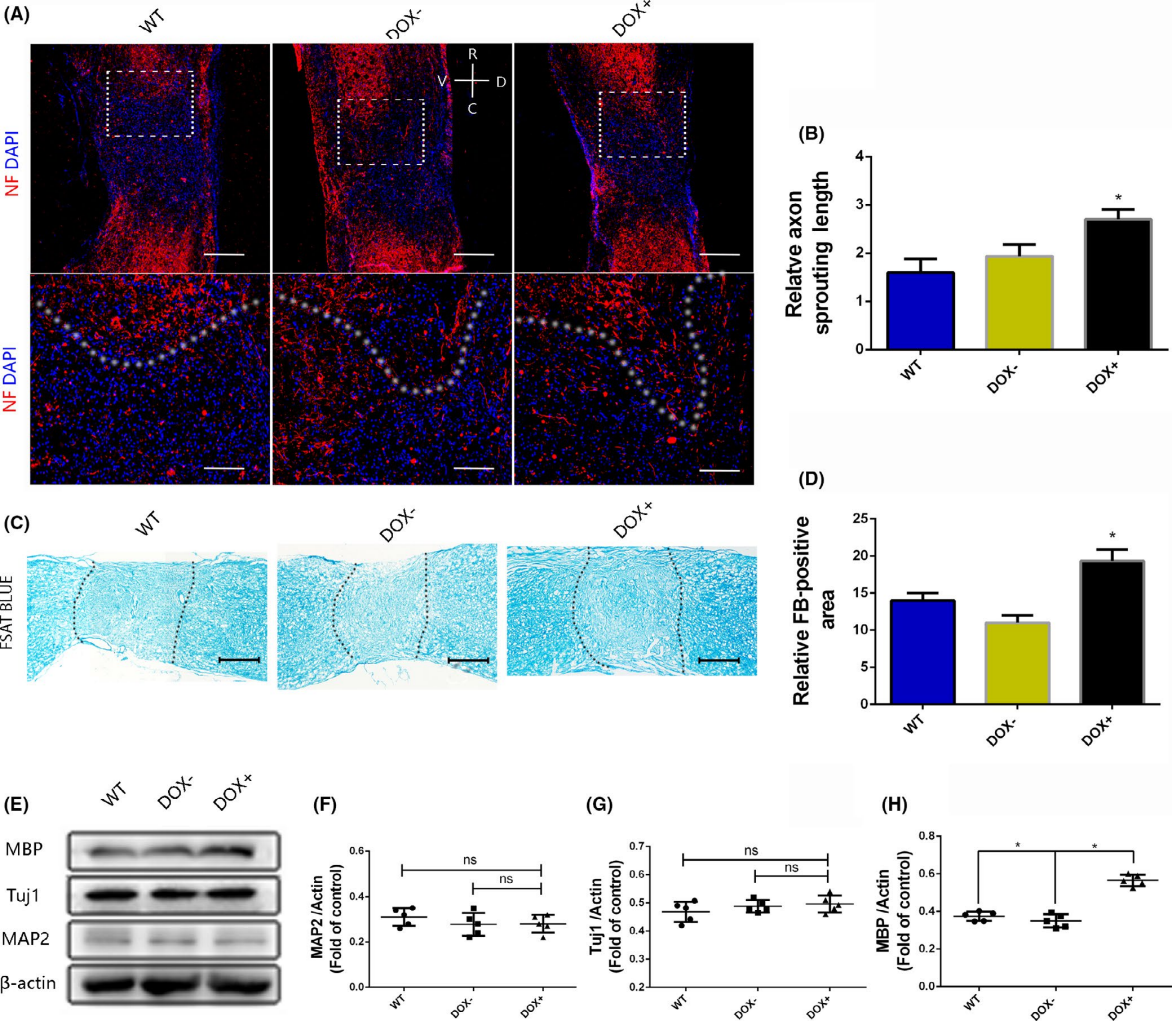
Remyelination is improved after the OCT4 and KLF4 transcription factors are overexpressed in vivo. (A, B) NF‐200 staining of spinal cord at 4 wk after SCI (R represents rostral, C represents caudal, V represents ventral, and D represents dorsal, scale bar, 500 or 200 μm); (C, D) fast blue staining of the spinal cord at 4 wk after SCI, scale bar, 50 μm); (E‐H) the protein expression levels of MAP2, Tuj1, and MBP in lesion area at 4 wk after SCI. Data were expressed as the mean ± SEM, one‐way ANOVA with Newman‐Keuls post hoc test, *P < .05, n = 5 per group

To explore the changes that occur in neural tissue after the overexpression of the transcription factors OCT4 and KLF4, we evaluated some neural markers related to axonal sprouting and remyelination. We discovered that some axons exhibited outgrowth into the lesion center and that these axons were longer in the DOX+ group than in the other two groups, as shown by immunostaining for NF‐200 around the lesion center (Figure 2A,B). Meanwhile, MAP2 and Tuj1 protein expression, as determined by Western blotting, was similar among the three groups (Figure 2E‐G). Additionally, we performed fast blue staining on spinal cord samples, and the DOX+ group showed more myelin sheaths than the other two groups (Figure 2C,D). MBP protein expression, as determined by Western blotting, was higher in the DOX+ group than in the other two groups (*P* < .05) (Figure 2E,H). These results indicated that remyelination was improved after the overexpression of the transcription factors OCT4 and KLF4. Remyelination improvement combined with CSPGs reduction may contribute to motor function recovery.

### 3.3 Overexpression of the transcription factors OCT4 and KLF4 enhances motor function after spinal cord injury

**FIGURE 3:**
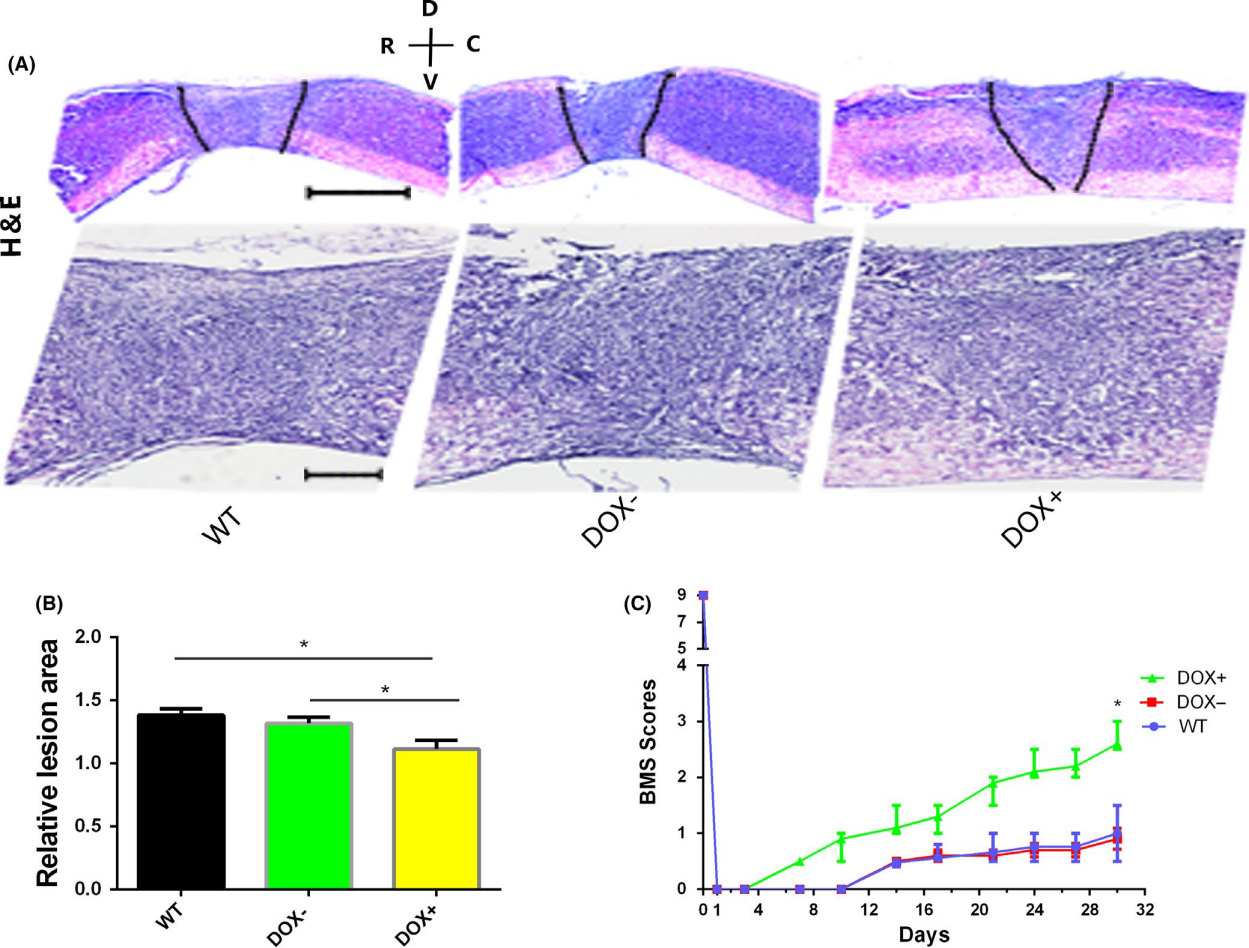
Histological examination of the spinal cord and a motor function test. (A) Hematoxylin and eosin (H&E) staining of the spinal cord 30 d after SCI (R represents rostral, C represents caudal, V represents ventral, and D represents dorsal, scale bar, 200 or 50 μm); (B) relative lesion area of part A, DOX+ vs DOX−, *P < .05; DOX+ vs WT, *P < .05, data were expressed as mean ± SEM, one‐way ANOVA with Newman‐Keuls post hoc test, n = 5 per group; (C) BMS scores of the individuals in three groups as determined every day after spinal cord injury induction. Data ware presented as median ± interquartile range, nonparametric *t* test, *P < .05, n = 5 per group

After 30 days of investigation, we found that mice in the DOX+ group had higher BMS scores than those in the WT and DOX− groups (*P* < .05) (Figure 3C). Histological H&E staining showed a smaller lesion area in the DOX+ group than in the other two groups (Figure 3A,B). These results indicated that the overexpression of OCT4 and KLF4 TFs can improve motor function after SCI. As a previous study demonstrated that OCT4 can induce fibroblasts to differentiate into oligodendrocyte progenitor cells and improve functional recovery, the effect of overexpression of the OCT4 and KLF4 TFs on the damaged central nervous system may be positive. Motor function improvement may be associated with motor neuron regeneration, remyelination, glial scar tissue reduction, and inflammatory response inhibition.^32^ Therefore, we investigated these aspects and revealed substantial changes that resulted from the overexpression of the “O‐K” transcription factors; however, the inflammatory response was not different between the DOX+ and control groups (data not shown).

### 3.4 Astrocytes are reprogrammed into neural stem cell‐like cells, and the migration ability of astrocytes is inhibited after the overexpression of the transcription factors OCT4 and KLF4

**FIGURE 4:**
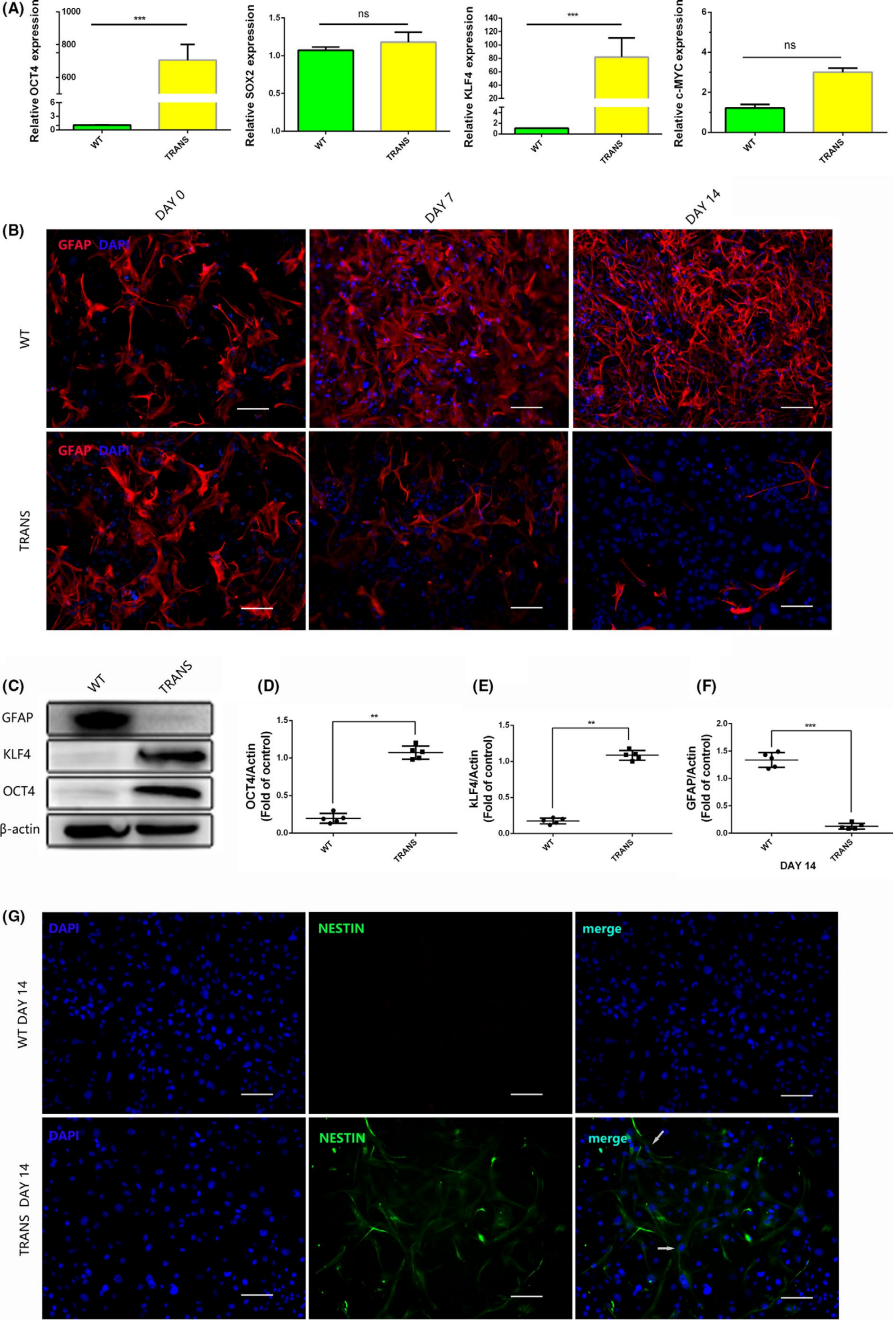
Astrocytes are reprogrammed into neural stem cell‐like cells after the transcription factors OCT4 and KLF4 were overexpressed in vitro. (A) Q‐PCR results of four transcription factors in astrocytes cultured for 3 d in Dox‐containing medium; (B) GFAP staining of astrocytes cultured in Dox‐containing medium for 0, 7, and 14 d, scale bar, 200 μm; (C‐F) protein expression levels of OCT4, KLF4 and GFAP were measured by Western blot analysis at day 14; (G) immunofluorescence stain of Nestin of astrocytes at day 14, scale bar, 200 μm. Data were expressed as mean ± SEM, unpaired Student's *t* test, **P < .01, ***P < .001, n = 5 per group

**FIGURE 5:**
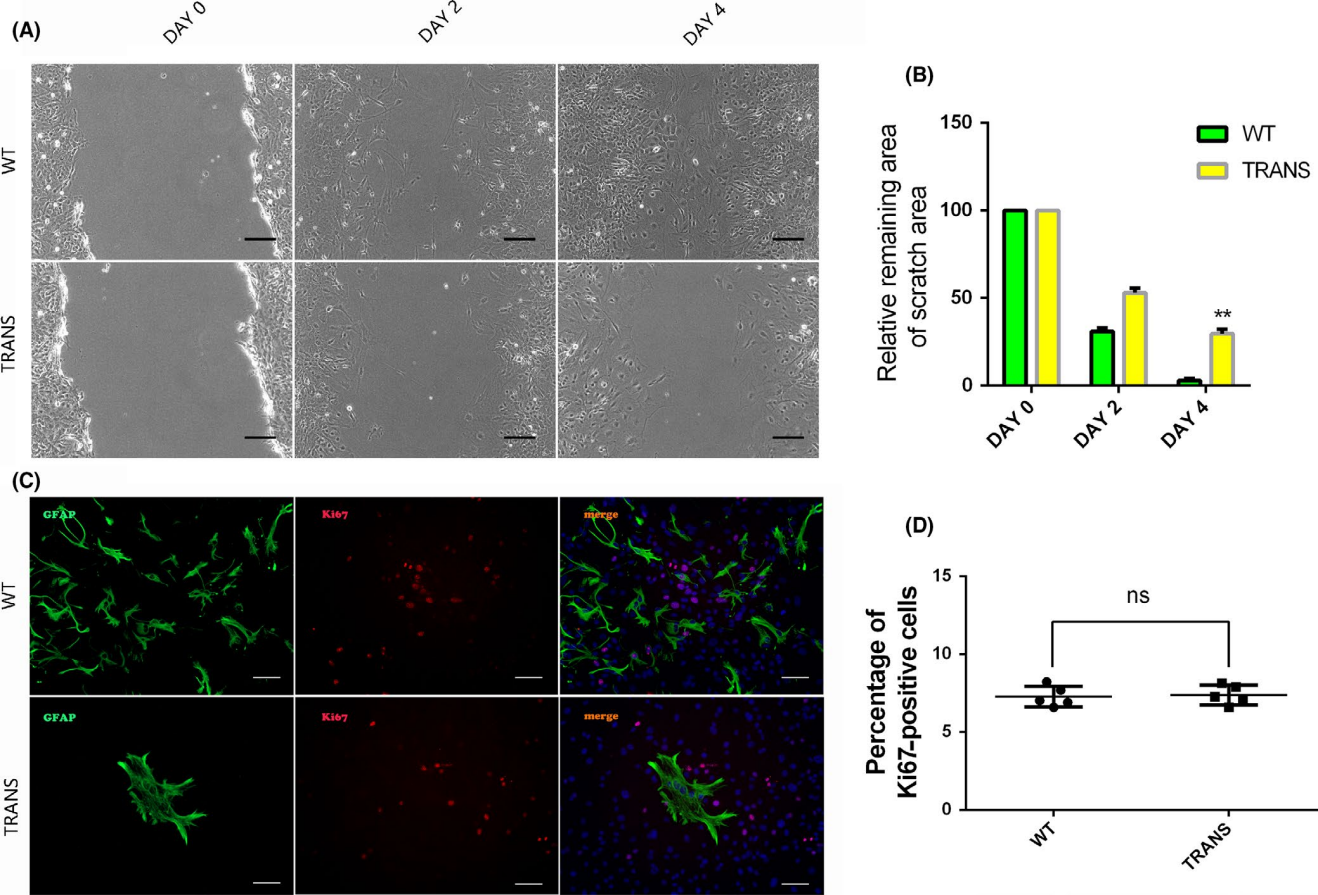
The migration ability of astrocytes is inhibited after the transcription factors OCT4 and KLF4 were overexpressed in vitro. (A) Cell scratch assay of astrocyte in vitro at day 0, 2, and 4, scale bar, 50 μm; (B) quantification of part A; (C) GFAP and Ki67 double staining after cultured with doxycycline at day 14; (D) quantification of part C, scale bar, 200 μm. Data were expressed as mean ± SEM, **P < .01, unpaired Student's *t* test, n = 5 per group

To reveal the mechanism underlying this phenomenon, we also designed an in vitro study of astrocytes. The q‐PCR and Western blotting results demonstrated that astrocytes in the TRANS group cultured in doxycycline‐containing medium overexpressed the transcription factors OCT4 and KLF4 (Figure 4A,C‐E). Interestingly, we discovered that the integrated density of GFAP was higher in the TRANS group than in the WT group on days 7 and 14 (*P* < .01). We tested GFAP protein expression on day 14, and the result was the same as the immunofluorescence result (Figure 4B,C,F). These results suggested that the proliferative ability of astrocytes was significantly inhibited or that astrocytes were reprogrammed into other types of cells. Accordingly, we performed staining for Nestin, which is a neural stem cell marker, on day 14. Interestingly, we found that in the TRANS group, there were some Nestin‐positive cells (6.56 ± 1.22%) (Figure 4G). In addition, Western blotting analysis showed that SOX2 protein expression level was higher than control group after overexpression of OCT4 and KLF4 (Figure S1A,B). These data indicated that astrocytes were reprogrammed into neural stem cell‐like cell (with nestin and sox2 expression). Furthermore, a cell scratch assay was used to test migration ability of astrocytes. In the WT group, the cells on each side of the scratch reached confluence after 4 days of culture in serum‐free medium, while the cells in the TRANS group did not (Figure 5A,B). The astrocyte transdifferentiation and reduction in the migration ability of astrocytes may contribute to CSPGs reduction and subsequent reconstruction.^33^ In addition, the proliferation ability of astrocyte was similar in two groups, showed by GFAP and Ki67 staining, (7.56 ± 1.45% vs 7.89 ± 1.68%) (Figure 5C,D).

### 3.5 The transcription factors OCT4 and KLF4 affect the biological behaviors of astrocytes by activating the Hippo/Yap pathway

**FIGURE 6:**
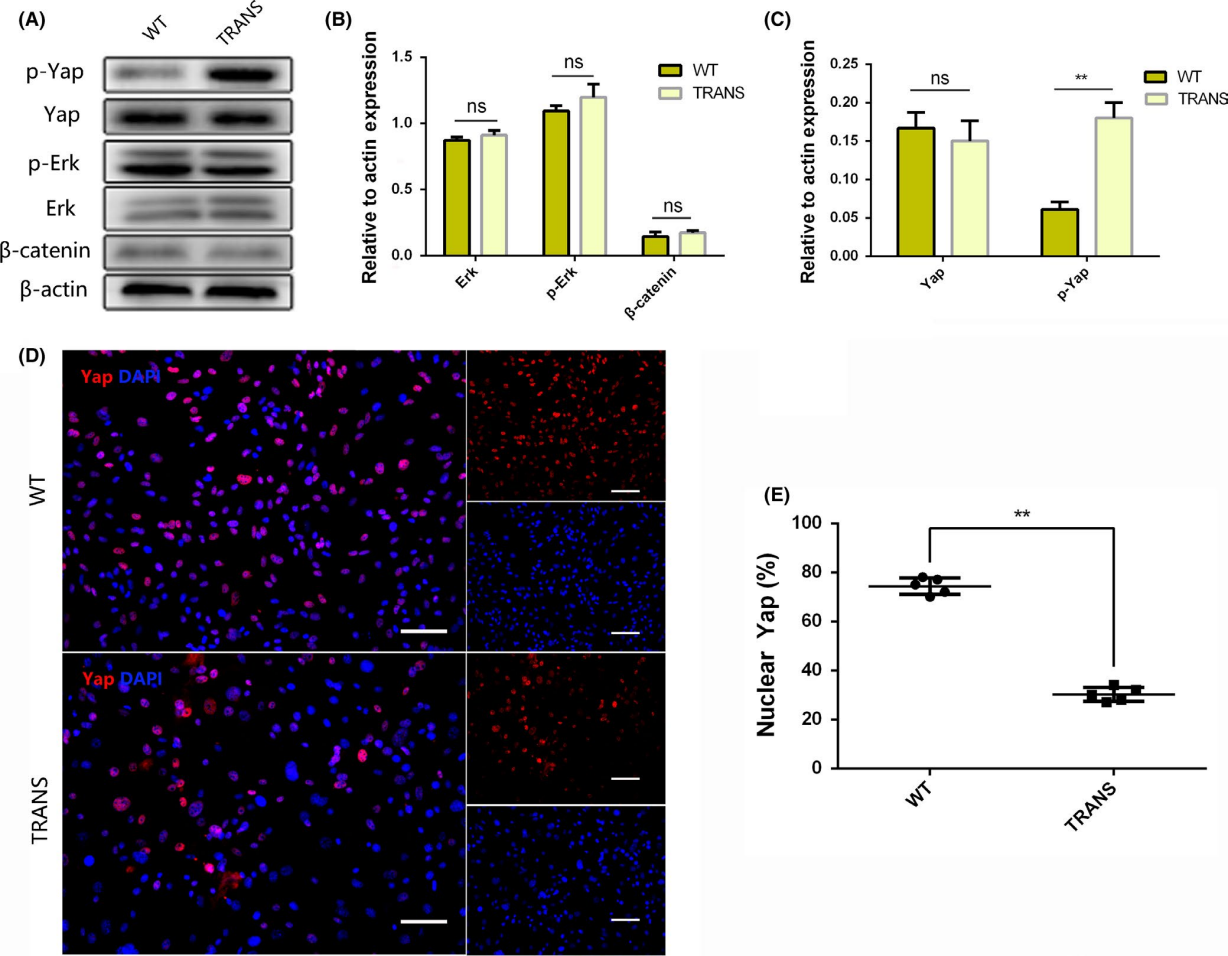
The transcription factors OCT4 and KLF4 affect the biological behaviors of astrocytes by activating the Hippo/Yap pathway. (A) The protein expression levels of Yap, p‐Yap, Erk, p‐Erk, and β‐catenin were measured by Western blot analysis at day 14 cultured on Dox‐containing medium; (B, C) quantification of part A; (D, E) Yap staining at day 14 cultured on Dox‐containing medium, scale bars, 200 μm. Data were expressed as mean ± SEM, unpaired Student's *t* test, **P < .01, n = 5 per group

**FIGURE 7:**
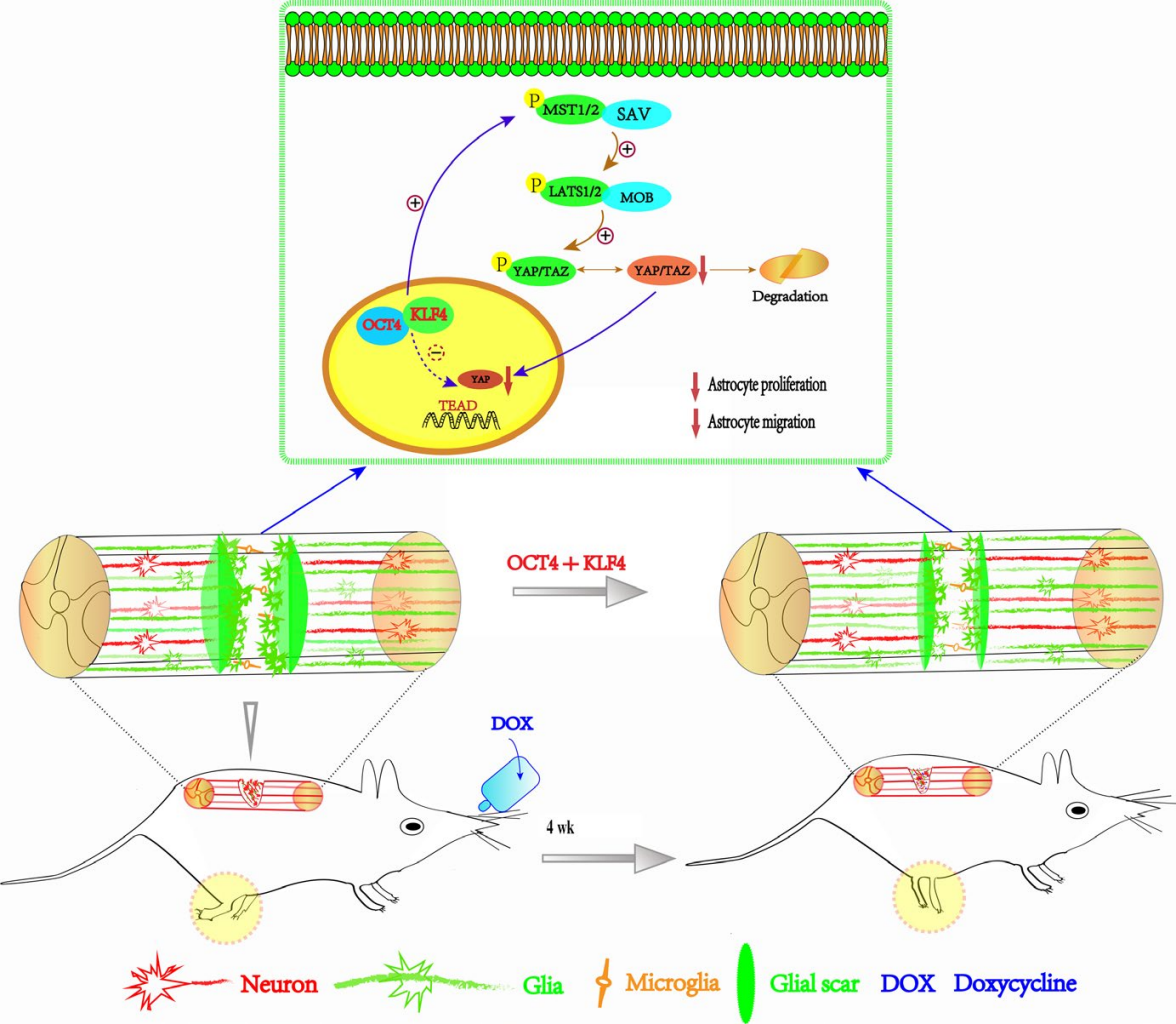
Schematics of the whole experiment. Overexpression of the transcription factors OCT4 and KLF4 could induce astrocyte reprogramming, which subsequently improves motor function after SCI, and the Hippo/Yap pathway plays a role

For finding the molecular mechanism behind the motor function changes, we then tested Wnt, MAPK, and Hippo/Yap pathway because of their roles in cell proliferation, differentiation, and migration. There was no difference in the Erk, p‐Erk, β‐catenin protein expression, but p‐Yap protein was much higher in TRANS group than in the WT group on day 14 (*P* < .01) (Figure 6A‐C). These data suggested that the Hippo/Yap pathway may be associated with the biological behaviors of astrocytes, as demonstrated in a previous study.^34^ Hence, we performed immunofluorescence staining for Yap at day 14, and the results showed that Yap protein expression level was lower in the nuclei of cells in the TRANS group than that in the WT group (25.33 ± 1.856 vs 69.33 ± 1.453), which indicated that the transcription factors OCT4 and KLF4 affected the biological behaviors of astrocytes through the activation of the Hippo/Yap pathway (*P* < .01) (Figure 6D,E). These data indicated that overexpression of the transcription factors OCT4 and KLF4 could induce astrocyte reprogramming, improving motor function after SCI (Figure 7).

## 4 DISCUSSION

Glial scars play dual roles in the reconstruction of an injured spinal cord. Scar tissue can restrict the injured area and inflammatory factor diffusion in the early phase after spinal cord injury hinders neurotropic factor infiltration and axonal outgrowth.^35^, ^36^, ^37^, ^38^ Our study provides evidence that the simultaneous overexpression of the transcription factors OCT4 and KLF4 reduces GFAP and CSPGs protein expression level in lesion site. Supporting this result, previous studies have demonstrated that OCT4 alone can induce mouse fibroblasts into oligodendrocyte progenitor cells and promote functional recovery.^24^ Moreover, KLF4 might be a regulator of proliferation and migration in neurons, blood, kidneys, and skin cells.^39^ Furthermore, Zarei‐Kheirabadi et al found that rat astrocytes were transdifferentiated into neural stem cells in vitro by ZFP521 or Sox2, and Wang et al confirmed that the p‐53 pathway could improve the reprogramming efficiency of the astrocytes to neuroblasts transition induced by Sox2.^40^, ^41^ In addition to these findings, the OCT4 and KLF4 TFs may be significant regulators of the biological behaviors of astrocytes by inducing cellular reprogramming. Specifically, our data showed that the porosity of the scar tissue was increased significantly by the inhibition of CSPGs secretion and hypertrophy‐activated astrocytes. These changes may facilitate axonal outgrowth and neurotrophic factor filtration and promote axonal regeneration.

Interestingly, we discovered that mice overexpressing the transcription factors OCT4 and KLF4 had higher BMS scores 30 days after SCI. Previous studies suggested that regenerated axons that connected properly with nerve fiber bundles across the lesion site were relevant to motor functional recovery and may be associated with inflammatory response regulation and astrocytic scar reduction.^42^ Therefore, our group focused on neuronal regeneration, and the results demonstrated that some of the axons sprouted into the lesion site after overexpression of the transcription factors OCT4 and KLF. However, there was no significant difference in the expression of neuron‐associated proteins between the experimental and control groups, a finding that may be related to the ability of the transcription factor KLF4 to regulate neurons.^39^ In addition, we discovered that remyelination was promoted in the injury site, which combined with CSPGs reduction and it may contribute to enhancement of motor ability.

To investigate the underlying mechanism of CSPGs reduction and remyelination after the overexpression of the transcription factors OCT4 and KLF4, we isolated astrocytes from the transgenic mice and cultured them in the proper medium. We found that GFAP expression in astrocytes was inhibited significantly in a time‐dependent manner. In addition, the migration ability of the astrocytes was suppressed, as demonstrated by the cell scratch assay. Two biological behaviors of the astrocytes were inhibited in vitro after the overexpression of the “O‐K” TFs, a finding consistent with the in vivo results. Notably, we then found that some astrocytes were reprogrammed into neural stem cell‐like cells, which expressed nestin and sox2 protein, a finding consistent with a previous study that demonstrated that OCT4 alone can induce the cellular reprogramming of fibroblasts or neural stem cells.^24^, ^43^ Astrocyte‐reprogrammed neural stem cell‐like cells may differentiate into specific cell subtypes to promote remyelination and subsequent rehabilitation after SCI. In addition, existing studies demonstrated that the Wnt, MAPK, and Hippo/Yap pathways are associated with cell proliferation, migration, and other significant biological behaviors.^44^, ^45^, ^46^ Thus, we hypothesized that these pathways may also play a role in regulating the biological behaviors of astrocytes. Interestingly, the data indicated that the Hippo/Yap signaling pathway was activated, as demonstrated by a previous study.^34^ Throughout the whole study, although no evidence of tumor was found, it is possible that tumors may develop in the long term.

In conclusion, our present study was the first to investigate the effect of overexpression of the transcription factors OCT4 and KLF4 on injured spinal cords. We discovered that the transcription factors OCT4 and KLF4 induced astrocyte reprogramming, improving remyelination and functional recovery. These results provide novel evidence that the OCT4 and KLF4 TFs can improve motor function after SCI in mice. Additionally, next work is needed to overexpress OCT4 and KLF4 alone to explore the involvement of each and potential synergistic effect and to find proper ways to enhance the efficiency of astrocyte reprogramming after spinal cord injury.

## CONFLICT OF INTEREST

The authors report no declaration of interest.

## AUTHORS' CONTRIBUTIONS

HXP and WCG designed the research and drafted the manuscript. LCZ, ZXP, WJK, and XKS performed the in vitro experiment. SJW, HB, CF, and YB performed the animal experiment. GZ, YLW, YC, and SKS collected the data and statistical analyses. CQX and LFC revised the manuscript, statistical analyses, and figures. All authors read and approved the final manuscript.

## Supporting information

Fig S1

## References

[1] Tran AP , Warren PM , Silver J . The biology of regeneration failure and success after spinal cord injury. Physiol Rev. 2018;7:881‐917. 10.1152/physrev.00017.2017 PMC5966716 29513146

[2] Karimi‐Abdolrezaee S , Billakanti R . Reactive astrogliosis after spinal cord injury‐beneficial and detrimental effects. Mol Neurobiol. 2012;46:251‐264. 22684804 10.1007/s12035-012-8287-4

[3] Ahuja CS , Wilson JR , Nori S , et al. Traumatic spinal cord injury. Nat Rev Dis Primers. 2017;3:17018. 28447605 10.1038/nrdp.2017.18

[4] Bellver‐Landete V , Bretheau F , Mailhot B , et al. Microglia are an essential component of the neuroprotective scar that forms after spinal cord injury. Nat Commun. 2019;10:518. 30705270 10.1038/s41467-019-08446-0 PMC6355913

[5] Silver J . The glial scar is more than just astrocytes. Exp Neurol. 2016;286:147‐149. 27328838 10.1016/j.expneurol.2016.06.018

[6] Adams KL , Gallo V . The diversity and disparity of the glial scar. Nat Neurosci. 2018;21:9‐15. 29269757 10.1038/s41593-017-0033-9 PMC5937232

[7] Bradbury EJ , Burnside ER . Moving beyond the glial scar for spinal cord repair. Nat Commun. 2019;10:3879. 31462640 10.1038/s41467-019-11707-7 PMC6713740

[8] Silver J , Miller JH . Regeneration beyond the glial scar. Nat Rev Neurosci. 2004;5:146‐156. 14735117 10.1038/nrn1326

[9] Renault‐Mihara F , Mukaino M , Shinozaki M , et al. Regulation of RhoA by STAT3 coordinates glial scar formation. J Cell Biol. 2017;216:2533‐2550. 28642362 10.1083/jcb.201610102 PMC5551705

[10] Lang BT , Cregg JM , DePaul MA , et al. Modulation of the proteoglycan receptor PTPsigma promotes recovery after spinal cord injury. Nature. 2015;518:404‐408. 25470046 10.1038/nature13974 PMC4336236

[11] Bradbury EJ , Moon LD , Popat RJ , et al. Chondroitinase ABC promotes functional recovery after spinal cord injury. Nature. 2002;416(6881):636‐640. 11948352 10.1038/416636a

[12] Thompson RE , Pardieck J , Smith L , et al. Effect of hyaluronic acid hydrogels containing astrocyte‐derived extracellular matrix and/or V2a interneurons on histologic outcomes following spinal cord injury. Biomaterials. 2018;162:208‐223. 29459311 10.1016/j.biomaterials.2018.02.013 PMC5851469

[13] Takahashi K , Yamanaka S . Induction of pluripotent stem cells from mouse embryonic and adult fibroblast cultures by defined factors. Cell. 2006;126:663‐676. 16904174 10.1016/j.cell.2006.07.024

[14] Taguchi J , Yamada Y . In vivo reprogramming for tissue regeneration and organismal rejuvenation. Curr Opin Genet Dev. 2017;46:132‐140. 28779646 10.1016/j.gde.2017.07.008

[15] Kim J , Efe JA , Zhu S , et al. Direct reprogramming of mouse fibroblasts to neural progenitors. Proc Natl Acad Sci USA. 2011;108:7838‐7843. 21521790 10.1073/pnas.1103113108 PMC3093517

[16] Son E , Ichida J , Wainger B , et al. Conversion of mouse and human fibroblasts into functional spinal motor neurons. Cell Stem Cell. 2011;9:205‐218. 21852222 10.1016/j.stem.2011.07.014 PMC3188987

[17] Caiazzo M , Dell'Anno MT , Dvoretskova E , et al. Direct generation of functional dopaminergic neurons from mouse and human fibroblasts. Nature. 2011;476:224‐227. 21725324 10.1038/nature10284

[18] Ambasudhan R , Talantova M , Coleman R , et al. Direct reprogramming of adult human fibroblasts to functional neurons under defined conditions. Cell Stem Cell. 2011;9:113‐118. 21802386 10.1016/j.stem.2011.07.002 PMC4567246

[19] Gao X , Wang X , Xiong W , Chen J . In vivo reprogramming reactive glia into iPSCs to produce new neurons in the cortex following traumatic brain injury. Sci Rep. 2016;6:22490. 26957147 10.1038/srep22490 PMC4783661

[20] Niu W , Zang T , Zou Y , et al. In vivo reprogramming of astrocytes to neuroblasts in the adult brain. Nat Cell Biol. 2013;15:1164‐1175. 24056302 10.1038/ncb2843 PMC3867822

[21] Su Z , Niu W , Liu ML , Zou Y , Zhang CL . In vivo conversion of astrocytes to neurons in the injured adult spinal cord. Nat Commun. 2014;5:3338. 24569435 10.1038/ncomms4338 PMC3966078

[22] Doeser MC , Scholer HR , Wu G . Reduction of fibrosis and scar formation by partial reprogramming in vivo. Stem Cells. 2018;36:1216‐1225. 29761584 10.1002/stem.2842

[23] Moore DL , Blackmore MG , Hu Y , et al. KLF family members regulate intrinsic axon regeneration ability. Science. 2009;326:298‐301. 19815778 10.1126/science.1175737 PMC2882032

[24] Kim JB , Lee H , Araúzo‐Bravo MJ , et al. Oct4‐induced oligodendrocyte progenitor cells enhance functional recovery in spinal cord injury model. EMBO J. 2015;34:2971‐2983. 26497893 10.15252/embj.201592652 PMC4687687

[25] Carey BW , Markoulaki S , Beard C , Hanna J , Jaenisch R . Single‐gene transgenic mouse strains for reprogramming adult somatic cells. Nat Methods. 2010;7:56‐59. 20010831 10.1038/nmeth.1410 PMC3048025

[26] Tan Z , Liu YU , Xi W , et al. Glia‐derived ATP inversely regulates excitability of pyramidal and CCK‐positive neurons. Nat Commun. 2017;8:13772. 28128211 10.1038/ncomms13772 PMC5290168

[27] Hsu J‐YC , Bourguignon LYW , Adams CM , et al. Matrix metalloproteinase‐9 facilitates glial scar formation in the injured spinal cord. J Neurosci. 2008;28:13467‐13477. 19074020 10.1523/JNEUROSCI.2287-08.2008 PMC2712293

[28] Zhou X , Wang J , Huang X , et al. Injectable decellularized nucleus pulposus‐based cell delivery system for differentiation of adipose‐derived stem cells and nucleus pulposus regeneration. Acta Biomater. 2018;81:115‐128. 30267879 10.1016/j.actbio.2018.09.044

[29] Lu P , Wang Y , Graham L , et al. Long‐distance growth and connectivity of neural stem cells after severe spinal cord injury. Cell. 2012;150:1264‐1273. 22980985 10.1016/j.cell.2012.08.020 PMC3445432

[30] Basso DM , Fisher LC , Anderson AJ , Jakeman LB , Mctigue DM , Popovich PG . Basso Mouse Scale for locomotion detects differences in recovery after spinal cord injury in five common mouse strains. J Neurotrauma. 2006;23:635. 16689667 10.1089/neu.2006.23.635

[31] Hesp ZC , Yoseph RY , Suzuki R , et al. Proliferating NG2‐cell‐dependent angiogenesis and scar formation alter axon growth and functional recovery after spinal cord injury in mice. J Neurosci. 2018;38:1366‐1382. 29279310 10.1523/JNEUROSCI.3953-16.2017 PMC5815343

[32] Selzer ME . Promotion of axonal regeneration in the injured CNS. Lancet Neurol. 2003;2:157‐166. 12849237 10.1016/s1474-4422(03)00322-3

[33] O'Shea TM , Burda JE , Sofroniew MV . Cell biology of spinal cord injury and repair. J Clin Investig. 2017;127:3259‐3270. 28737515 10.1172/JCI90608 PMC5669582

[34] Deng W , Shao F , He Q , et al. EMSCs build an all‐in‐one niche via cell‐cell lipid raft assembly for promoted neuronal but suppressed astroglial differentiation of neural stem cells. Adv Mater. 2019;31:e1806861. 30633831 10.1002/adma.201806861

[35] Cregg JM , DePaul MA , Filous AR , Lang BT , Tran A , Silver J . Functional regeneration beyond the glial scar. Exp Neurol. 2014;253:197‐207. 24424280 10.1016/j.expneurol.2013.12.024 PMC3951813

[36] Okada S , Nakamura M , Katoh H , et al. Conditional ablation of Stat3 or Socs3 discloses a dual role for reactive astrocytes after spinal cord injury. Nat Med. 2006;12:829‐834. 16783372 10.1038/nm1425

[37] Anderson MA , Burda JE , Ren Y , et al. Astrocyte scar formation aids central nervous system axon regeneration. Nature. 2016;532:195‐200. 27027288 10.1038/nature17623 PMC5243141

[38] Yiu G , He Z . Glial inhibition of CNS axon regeneration. Nat Rev Neurosci. 2006;7:617‐627. 16858390 10.1038/nrn1956 PMC2693386

[39] Ghaleb AM , Yang VW . Kruppel‐like factor 4 (KLF4): what we currently know. Gene. 2017;611:27‐37. 28237823 10.1016/j.gene.2017.02.025 PMC5391259

[40] Wang LL , Su Z , Tai W , Zou Y , Xu XM , Zhang CL . The p53 pathway controls SOX2‐mediated reprogramming in the adult mouse spinal cord. Cell Rep. 2016;17:891‐903. 27732862 10.1016/j.celrep.2016.09.038 PMC5094368

[41] Zarei‐Kheirabadi M , Hesaraki M , Kiani S , Baharvand H . In vivo conversion of rat astrocytes into neuronal cells through neural stem cells in injured spinal cord with a single zinc‐finger transcription factor. Stem Cell Res Ther. 2019;10:380. 31842989 10.1186/s13287-019-1448-x PMC6916443

[42] Tohda C , Kuboyama T . Current and future therapeutic strategies for functional repair of spinal cord injury. Pharmacol Ther. 2011;132:57‐71. 21640756 10.1016/j.pharmthera.2011.05.006

[43] Kim JB , Greber B , Araúzo‐Bravo MJ , et al. Direct reprogramming of human neural stem cells by OCT4. Nature. 2009;461:649‐653. 19718018 10.1038/nature08436

[44] Misra JR , Irvine KD . The hippo signaling network and its biological functions. Annu Rev Genet. 2018;52:65‐87. 30183404 10.1146/annurev-genet-120417-031621 PMC6322405

[45] Clevers H . Wnt/beta‐catenin signaling in development and disease. Cell. 2006;127:469‐480. 17081971 10.1016/j.cell.2006.10.018

[46] Sun Y , Liu WZ , Liu T , Feng X , Yang N , Zhou HF . Signaling pathway of MAPK/ERK in cell proliferation, differentiation, migration, senescence and apoptosis. J Recept Signal Transduct Res. 2015;35:600‐604. 26096166 10.3109/10799893.2015.1030412

