# Supplementary material for: Overexpression of the transcription factors OCT4 and KLF4 improves motor function after spinal cord injury
Source: CNS Neurosci Ther. 2020 May 25;26(9):940–51. doi: 10.1111/cns.13390 (PMC7415207; doi:10.1111/cns.13390)
Supplement: Supplementary file 1 — Fig S1 [file CNS-26-940-s001.pdf]

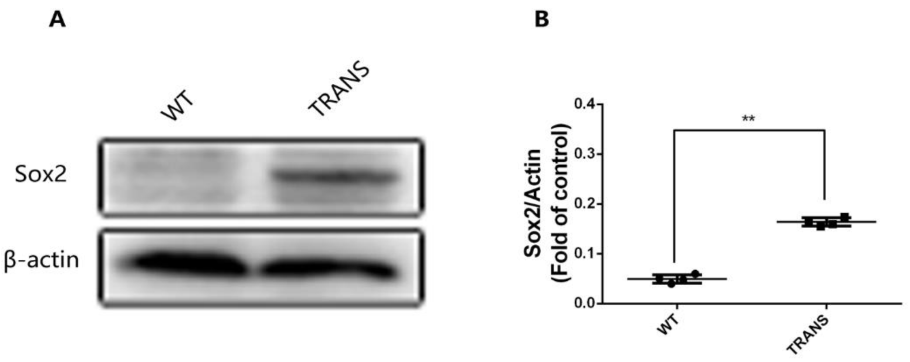

Supplementary Fig.1 Sox2 expression level increased after overexpression of OCT4 and KLF4. A) Western blotting result at day 14 induced with doxycycline; B) Qualification of part A. Data were expressed as mean ± SEM, unpaired Student's t-test, **$P < 0.01$, n= 4 per group.
